# Promising potential of new generation translocator protein tracers providing enhanced contrast of arthritis imaging by positron emission tomography in a rat model of arthritis

**DOI:** 10.1186/ar4509

**Published:** 2014-03-14

**Authors:** Yoony YJ Gent, Karin Weijers, Carla FM Molthoff, Albert D Windhorst, Marc C Huisman, Michael Kassiou, Gerrit Jansen, Adriaan A Lammertsma, Conny J van der Laken

**Affiliations:** 1 Department of Rheumatology, VU University Medical Center, De Boelelaan 1117, Amsterdam 1081 HV, The Netherlands; 2 Department of Radiology & Nuclear Medicine, VU University Medical Center, De Boelelaan 1117, Amsterdam 1081 HV, The Netherlands; 3 School of Chemistry, The University of Sydney, Sydney NSW 2006, Australia; 4 Brain and Mind Research Institute, Sydney NSW 2050, Australia; 5 Discipline of Medical Radiation Sciences, The University of Sydney, Sydney NSW 2006, Australia

## Abstract

**Introduction:**

Early diagnosis of and subsequent monitoring of therapy for rheumatoid arthritis (RA) could benefit from detection of (sub)clinical synovitis. Imaging of (sub)clinical arthritis by targeting the translocator protein (TSPO) on activated macrophages is feasible using *(R)-*[^11^C] PK11195-based positron emission tomography (PET), but clinical applications are limited by background uptake in peri-articular bone/bone marrow. The purpose of the present study was to evaluate two other TSPO ligands with potentially lower background uptake in neurological studies, [^11^C]DPA-713 and [^18^F]DPA-714, in a rat model of arthritis.

**Methods:**

TSPO binding of DPA-713, DPA-714 and PK11195 were assessed by *in vitro* competition studies with [^3^H]DPA-713 using human macrophage THP-1 cells and CD14^+^ monocytes from healthy volunteers. *In vivo* studies were performed in rats with methylated bovine serum albumin-induced knee arthritis. Immunohistochemistry with anti-TSPO antibody was performed on paraffin-embedded sections. Rats were imaged with [^11^C]DPA-713 or [^18^F]DPA-714 PET, followed by *ex vivo* tissue distribution studies. Results were compared with those obtained with the tracer *(R)-*[^11^C]PK11195, the established ligand for TSPO.

**Results:**

In THP-1 cells, relative TSPO binding of DPA-713 and DPA-714 were 7-fold and 25-fold higher, respectively, than in PK11195. Comparable results were observed in CD14^+^ monocytes from healthy volunteers. In the arthritis rat model, immunohistochemistry confirmed the presence of TSPO-positive inflammatory cells in the arthritic knee. PET images showed that uptake of [^11^C]DPA-713 and [^18^F]DPA-714 in arthritic knees was significantly increased compared with contralateral knees and knees of normal rats. Uptake in arthritic knees could be largely blocked by an excess of PK11195. [^11^C]DPA-713 and [^18^F]DPA-714 provided improved contrast compared with *(R)*-[^11^C]PK11195, as was shown by significantly higher arthritic knee-to-bone ratios of [^11^C]DPA-713 (1.60 ± 0.31) and [^18^F]DPA-714 (1.55 ± 0.10) compared with *(R)*-[^11^C]PK11195 (1.14 ± 0.19).

**Conclusions:**

[^11^C]DPA-713 and [^18^F]DPA-714 clearly visualized arthritis and exhibited lower (peri-articular) bone/bone marrow uptake than *(R)-*[^11^C]PK11195. These features merit further investigation of these tracers for early diagnosis and therapy monitoring of RA in a clinical setting.

## Introduction

Macrophages play a central role in the pathogenesis of rheumatoid arthritis (RA) by driving synovial inflammation and subsequent tissue destruction [1]. Macrophages infiltrate the synovium early in the course of the disease with both number and level of macrophage activation correlating with clinical disease activity [2]. Visualization of synovial macrophages using specific positron emission tomography (PET) tracers could, therefore, offer the possibility of both early diagnosis [3] and sensitive monitoring of disease [4].

The translocator protein (TSPO, formerly known as the peripheral benzodiazepine receptor) is primarily localized in the outer mitochondrial membrane, where it has a function in cholesterol transport as a rate-limiting step in steroid biosynthesis [5]. Increased TSPO expression has been associated with an activated state of macrophages and microglia [6], based on which TSPO has been recognized as a biomarker for inflammatory diseases [7].

The prototypical radioligand for PET imaging of the TSPO is *(R)*-[^11^C]PK11195 (1-[2-chlorophenyl]-*N*-methyl-*N*-[1-methyl-propyl]-3-isoquinoline carboxamide) [8]. Recent studies have demonstrated that, although imaging of clinical and subclinical arthritis with *(R)*-[^11^C]PK11195 PET is promising, considerable background uptake of the tracer in peri-articular tissues (in particular bone/bone marrow) may hamper detection of subtle changes in macrophage infiltration and inflammation [3,9].

Previous *(R)*-[^11^C]PK11195 studies in neuroinflammation have shown a relatively low specific-to-nonspecific ratio, possibly due to its high lipophilicity and low bioavailability [10]. This has initiated research in developing next generation TSPO tracers that exhibit higher binding affinities and better specific-to-nonspecific binding ratios than *(R)*-[^11^C]PK11195 [11]. Two candidate compounds that meet this profile include [^11^C]DPA-713 (*N*,*N*-diethyl-2-(4-methoxyphenyl)-5,7-dimethylpyrazolo[1,5-*a*]pyrimidine-3-acetamide) and [^18^F]DPA-714 (*N,N*-diethyl-2-(2-[4-(2-fluoroethoxy)-phenyl]-5,7-dimethylpyrazolo[1,5-a]pyrimidin-3-yl)-acetamide) [12-14].

The potential of the DPA tracers for PET imaging of RA has not yet been reported. Therefore, the aim of this study was to evaluate [^11^C]DPA-713 and [^18^F]DPA-714 as novel candidate PET tracers in a rat model of RA, using a direct comparison to *(R)*-[^11^C]PK11195.

## Methods

### Synthesis of (***R***)-[^11^C]PK11195, [^11^C]DPA-713, [^18^F]DPA-714 and [^3^H]DPA-713

*(R)*-[^11^C]PK11195 was synthesized routinely with a radiochemical purity >98%, and a mean specific activity of 95.7 ± 28.4 GBq/μmole [15]. [^11^C]DPA-713 and [^18^F]DPA-714 were synthesized routinely with a radiochemical purity of >98% and >95% and a specific activity of >40 GBq/μmole and >75 GBq/μmole, respectively. One [^11^C]DPA-713 synthesis yielded a specific activity of 1.5 GBq/μmole, however, this did not affect *in vivo* results.

[^3^H]DPA-713 was synthesized with a radiochemical purity of 99.8% and a specific activity of 2.37 GBq/μmole. For a detailed description of [^11^C]DPA-713, [^18^F]DPA-714 and [^3^H]DPA-713 synthesis see Additional file 1.

### *In vitro* binding competition assay

*In vitro* TSPO binding competition assays were performed using the human monocytic-macrophage cell line THP-1, which expresses high TSPO levels [16] and freshly isolated human monocytes. THP-1 cells were grown in RPMI-1640 medium supplemented with 10% fetal calf serum, 2 mM L-glutamine and 100 μg/mL penicillin/streptomycin. Cells were harvested in the mid-log phase of growth and washed twice in ice-cold Hepes-buffered saline solution (pH 7.4) supplemented with 0.1% (w/v) BSA and finally resuspended in this buffer to a density of 1 × 10^6^ cells/mL. Next, 1 mL of this cell suspension was incubated in an Eppendorf tube with 10 pmol [^3^H]DPA-713 in the absence or presence of a 0.25- to 100-fold molar excess of unlabelled PK11195 (Sigma Aldrich Chemie BV, Zwijndrecht, The Netherlands), DPA-713 or DPA-714 (provided by M. Kassiou). Peripheral blood mononuclear cells were isolated from healthy volunteers by Ficoll density gradient centrifugation. CD14^+^ monocytes were positively selected with magnetic-activated cell sorting (MACS) magnetic beads (Miltenyi Biotec, Bergisch Gladbach, Germany) according to the manufacturer’s protocol. One mL of 10^6^ CD14^+^ cells were incubated with 10 pmol [^3^H]DPA-713 in the absence or presence of 20 or 50 pmol unlabelled PK11195, DPA-713 or DPA-714.

Following 30 minutes incubation at 4°C, THP-1 cells or CD14^+^ monocytes were centrifuged for one minute in an Eppendorf centrifuge (1,200 rpm). The supernatant was aspirated and the remaining fluid removed by cotton swabs. The cell pellet was then resuspended in 200 μL of H_2_O and transferred to a scintillation vial containing 5 mL Opti-Fluor (PerkinElmer, Waltham, Massachusetts, USA) scintillation fluid. The cell-associated amount of [^3^H]DPA-713 was assessed with a liquid scintillation analyzer (Tri-Carb 2800TR, PerkinElmer, Waltham, Massachusetts, USA). Samples containing a 1,000-fold molar excess of unlabelled PK11195 served as controls for specificity. Ethical approval was obtained from the ethics committee of the VU University Medical Center and informed consent was given by all healthy controls.

### Animals

Male Wistar rats (body weight approximately 200 to 300 g, Charles River International Inc, Sulzfeld, Germany) were fed with standard food (16% protein rodent diet, Harlan Laboratories Inc., Madison, WI, USA) and water *ad libitum*. Rats were housed in groups of four in standard laboratory cages and kept in an air-conditioned room at a temperature of approximately 21°C, a humidity level of about 50% and under a 12 hour light/12 hour dark cycle. Arthritic rats were scanned with *(R)*-[^11^C]PK11195 (n = 5), [^11^C]DPA-713 (n = 7) or [^18^F]DPA-714 (n = 6), which was followed by tissue distribution (n = 5, 7, 5, respectively). Two arthritic rats per tracer were used for TSPO binding blocking studies (that is, PET scanning and tissue distribution) with [^11^C]DPA-713 and [^18^F]DPA-714, and unlabelled PK11195. In addition, five normal (untreated) rats per tracer were used for tissue distribution studies with [^11^C]DPA-713 and [^18^F]DPA-714. Animal experiments were performed in accordance with the Dutch law on animal experimentation and the protocol was approved by the committee on animal experimentation of the VU University Medical Center.

### Induction of knee arthritis

Antigen-induced arthritis was elicited as described previously, but with some adaptations for optimal PET imaging of arthritis [17-20]. Subcutaneous immunization was performed at days 0 and 7 with 200 μL of a solution containing 50 mg mBSA (Sigma-Aldrich Chemie BV, Zwijndrecht, The Netherlands) dissolved in 1 mL 0.9% NaCl and emulsified in 1 mL complete Freund’s adjuvant (Sigma-Aldrich, Steinheim, Germany) and 1 mL (10^11^ particles) custom Bordetella pertussis antigen (Becton Dickinson, Breda, The Netherlands). Induction of mono-arthritis was achieved at day 20 by an intra-articular injection in the right knee with 60 μL of a solution of 10 mg mBSA in 1 mL 0.9% NaCl. At day 27, PET scans were performed, after which rats were sacrificed immediately for analysis of *ex vivo* tissue distribution of the tracers.

### Histopathology and immunohistochemistry

Both knees were dissected *in toto* and fixed (10% paraformaldehyde, 2% sucrose/phosphate buffered saline, pH 7.3) for seven days at 4°C. Bone tissue of the knees was decalcified for approximately seven weeks at 4°C in a solution with 123 mM sodium ethylenediaminetetraacetic acid (Na_2_-EDTA.2H_2_O, Merck, Darmstadt, Germany) (pH 7.2) and 113 nM NaOH (Sigma-Aldrich Chemie BV). The decalcification solution was regularly refreshed. After decalcification, knees were rinsed and processed for paraffin embedding. Sagittal sections (5 μm) from the center of the joint were used for immunohistochemical staining for the TSPO receptor using the rabbit anti-TSPO polyclonal antibody NP115 (kindly provided by Prof. Higuchi, National Institute of Radiological Sciences, Chiba, Japan) [21]. The immunohistochemical procedure was performed as follows: sections were deparaffinized and autoclaved for five minutes at 121°C in 0.01 M citrate buffer (pH 6.0) for antigen retrieval. This was followed by incubation with 0.3% H_2_O_2_ in methanol for 30 minutes at room temperature and overnight incubation at 4°C with 1:1,500 diluted NP155. Sections were washed extensively with a buffer containing 0.1 M Tris–HCl (pH 7.5), 0.15 M NaCl and 0.05% Tween20 before incubation with the secondary antibody (swine anti-rabbit, (DAKO, Glostrup, Denmark) E0353, 1:300 diluted) for 30 minutes at room temperature. Finally, the Vectastain ABC kit (Vector Laboratories, Burlingame, CA, USA) was used according to the manufacturer’s instructions. Peroxidase activity was detected using a solution of 3.3′-diaminobenzidine tetrahydrochloride containing 0.01% H_2_O_2_. Subsequently, sections were counterstained with hematoxylin, dehydrated and mounted. Negative controls were included by replacement of the primary antibody with 1% BSA/PBS. A Leica 4000B microscope and Leica digital camera DC500 (Microsystems B.V., Rijswijk, The Netherlands) were used to capture images.

### PET scanning protocol

Scanning was performed using a LSO/LYSO double layer ECAT High Resolution Research Tomograph (HRRT, CTI/Siemens, Knoxville, TN, USA): a small animal and human brain three-dimensional scanner with high spatial resolution (2.3 to 3.4 mm full width at half maximum) and high sensitivity [22]. Rats were anesthetized (approximately 2% isofluran with an oxygen flow of 0.6 to 0.8 L/min) before positioning in the HRRT. A static transmission scan (six minutes) using a rotating 740 MBq ^137^Cs point source was performed. A one hour emission scan was started at intravenous injection of 10.5 ± 2.9 MBq *(R)*-[^11^C]PK11195, 17.0 ± 3.7 MBq [^11^C]DPA-713 or 14.5 ± 3.7 MBq [^18^F]DPA-714 via a jugular vein canula. PET data were normalized and corrected for scatter, randoms, attenuation, decay and dead time and converted into 16 sinograms (5 × 5, 5 × 10, 3 × 15, 2 × 30, 2 × 60, 2 × 150, 2 × 300, 1 × 600, 2 × 900 s). Images were reconstructed using an iterative three-dimensional ordered subset expectation maximization (OSEM) with eight iterations and sixteen subsets. Resulting images had a matrix size of 256 × 256 × 207 voxels, each with a dimension of 1.21 × 1.21 × 1.21 mm^3^.

### PET image analysis

PET images were analyzed using AMIDE software (Amide’s a Medical Image Data Examiner, version 0.9.2) [23]. Fixed size ellipsoidal shaped regions of interest (ROI) (dimensions: 6.0 × 17.7 × 7.4 mm^3^) were manually drawn over the area of the left and right knees in the last frame of the image. ROIs were projected onto the dynamic image sequence, and time-activity curve (TAC) data were extracted. TACs were expressed as standardized uptake values (SUV): mean ROI radioactivity concentration normalized for injected dose and body weight. Uptake of *(R)*-[^11^C]PK11195, [^11^C]DPA-713 and [^18^F]DPA-714 in both knees was determined by averaging the SUV values obtained over the period from 50 to 60 minutes post-injection.

### *Ex vivo* tissue distribution studies

Immediately after each scan, that is, 60 minutes after intravenous injection of *(R)*-[^11^C]PK11195, [^11^C]DPA-713 or [^18^F]DPA-714, heart puncture of anesthetized rats was performed to collect blood samples. Next, rats were sacrificed by cervical dislocation, which was followed by excision and weighing of both knees (*in toto*), bone obtained from the right hind leg and tissue from various internal organs. Radioactivity present in blood and tissues (in percentage injected dose per gram of tissue: % ID/g) was determined using an LKB 1282 Compugamma CS gamma counter (LKB Wallac, Turku, Finland).

### *In vivo* blocking of TSPO binding

Tracer binding was investigated to verify that binding to the inflamed synovium was mediated through TSPO. To this end, blocking studies were performed in arthritic rats by pre-administration of 5 mg/kg unlabelled PK11195 (Sigma Aldrich Chemie BV) five minutes prior to the injection of [^11^C]DPA-713 or [^18^F]DPA-714. PET and *ex vivo* tissue distribution studies were performed as described above.

### Statistical analysis

Statistical tests were performed using IBM SPSS Statistics 20 (IBM, Armonk, NY, USA). A Wilcoxon signed rank (exact) test was used to determine differences in paired observations, for example, uptake of *(R)*-[^11^C]PK11195, [^11^C]DPA-713 or [^18^F]DPA-714 in arthritic versus contralateral knees. Mann–Whitney (exact) tests were performed to analyze differences in *(R)*-[^11^C]PK11195, [^11^C]DPA-713 or [^18^F]DPA-714 uptake in arthritic versus normal knees. Kruskal-Wallis (exact) tests were performed to determine differences between *(R)*-[^11^C]PK11195, [^11^C]DPA-713 and [^18^F]DPA-714 absolute uptake and tissue uptake ratios. If significance was found, Mann–Whitney (exact) tests with Bonferroni correction were used as *post-hoc* tests. Results are presented as mean ± standard deviation (SD) unless stated otherwise. *P*-values <0.05 were considered statistically significant.

## Results

### *In vitro* TSPO competition assay

**Figure 1:**
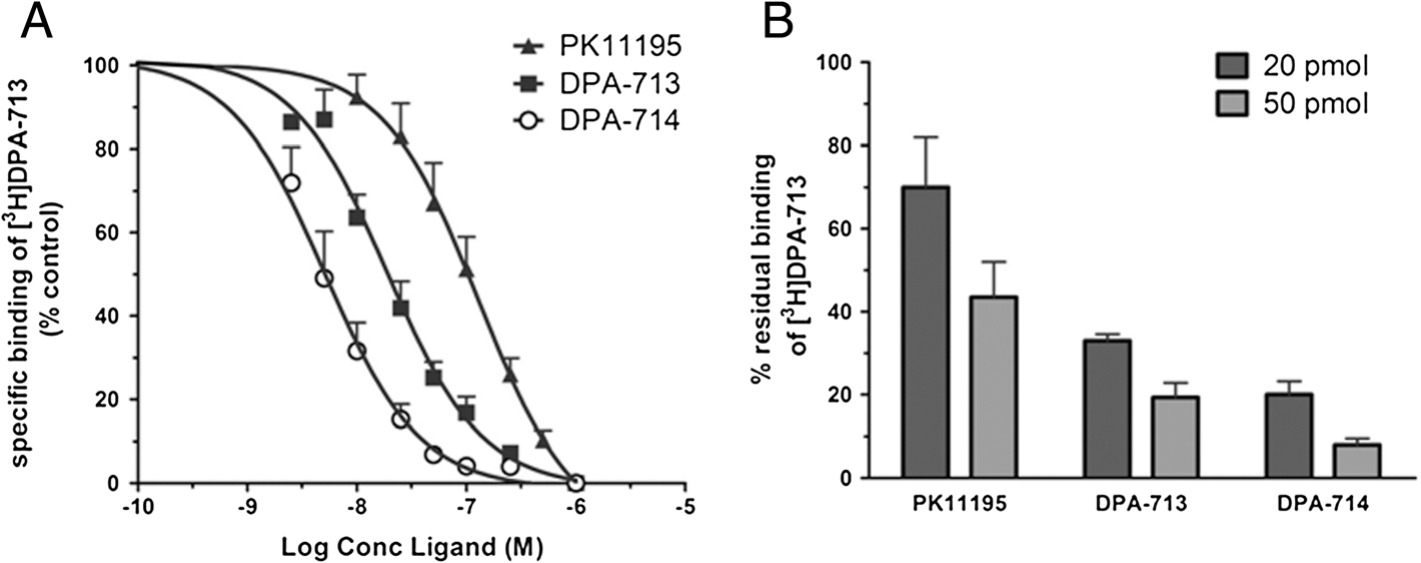
**[**^**3**^**H]DPA-713 TSPO binding competition studies. (A)** Displacement of [^3^H]DPA-713 TSPO binding by PK11195, DPA-713 and DPA-714 in human THP-1 cells. Cells were incubated with 10 nM [^3^H]DPA-713 in the presence of increasing concentrations of unlabelled PK11195, DPA-713 and DPA-714. Fifty percent displacement of [^3^H]DPA-713 was observed at 128 nM PK11195, 18.5 nM DPA-713, and 5.2 nM DPA-714. Results are presented as mean ± SEM of three separate experiments. **(B)** [^3^H]DPA-713 binding competition by unlabelled PK11195, DPA713 and DPA714 in CD14^+^ monocytes from healthy volunteers. Assay conditions were that 1 mL of 10^6^ CD14^+^ cells were incubated with 10 pmol [^3^H]DPA-713 in the absence or presence of 20 or 50 pmol unlabelled PK11195, DPA-713 or DPA-714. Residual binding of [^3^H]DPA-713 under these conditions is depicted. Addition of excess (2,000-fold molar excess) DPA-713 resulted in almost complete displacement of [^3^H]DPA-713 (that is, 0.3 ± 0.2% residual binding, results not shown). Mean [^3^H]DPA-713 binding capacity was 2.6 pmol/10^6^ CD14^+^ cells (range 1.3 to 5.1). Results are presented as mean ± SEM of three separate experiments. SEM, standard error of the mean; TSPO, translocator protein.

Figure 1 shows displacement of [^3^H]DPA-713 from TSPO in the presence of increasing concentrations of unlabelled DPA-713, DPA-714 and PK11195. In THP-1 cells it was demonstrated that for 50% displacement of [^3^H]DPA-713 binding to TSPO, 7-fold lower concentrations of DPA-713 and 25-fold lower concentrations of DPA-714 were needed compared with PK11195 (Figure 1A). Comparable results with the same ranking were observed in freshly isolated human CD14^+^ monocytes (Figure 1B). Thus, DPA-714 and DPA-713 showed higher relative binding compared with that of PK11195.

### Presence of synovial TSPO-positive cells in arthritic knees

**Figure 2:**
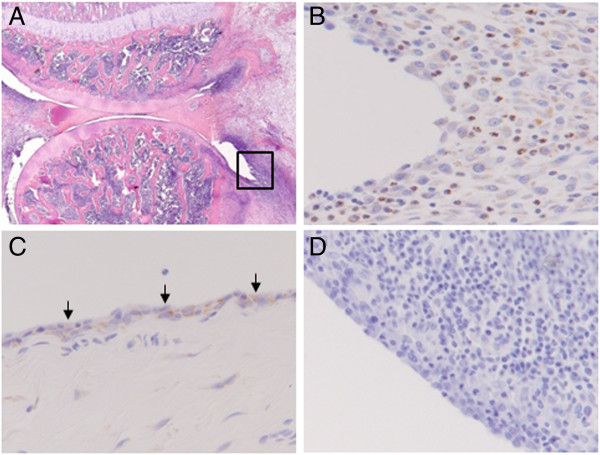
**Immunohistochemical staining for macrophages and TSPO. (A)** Hematoxylin and eosin staining of an arthritic knee. The square represents the region of the synovium as shown in panels **B** and **D** (magnification, 25x) **(B)** Immunohistochemical staining for TSPO (brown color) in arthritic knee (magnification, 200x). **(C)** Immunohistochemical staining for TSPO (brown color) in contralateral knee (magnification, 200x). Arrows indicate the synovial lining in which the TSPO-positive cells reside. **(D)** Tissue section of arthritic knee: negative control (magnification, 200x). TSPO, translocator protein.

Intra-articular injection of mBSA in the right knees of immunized rats provoked development of arthritis which was characterized by an influx of TSPO-positive cells in the synovium as shown by immunohistochemical staining (Figure 2B). Based on cell morphology, TSPO-positive cells were largely recognized as macrophages and neutrophils. In contrast, in the contralateral control knee only limited numbers of TSPO-positive cells were present (Figure 2C).

### PET studies

**Figure 3:**
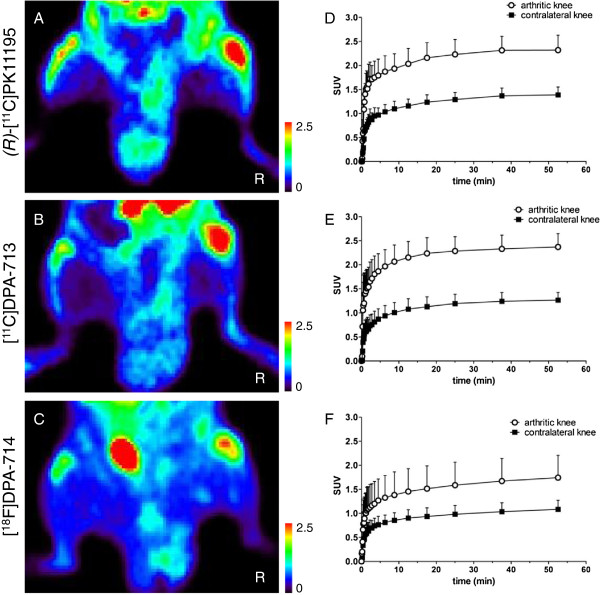
**[**^**11**^**C]DPA-713, [**^**18**^**F]DPA-714 and *(R)*-[**^**11**^**C]PK11195 PET images and corresponding TAC of arthritic and contralateral knees.** Representative examples of [^11^C]DPA-713, [^18^F]DPA-714 and *(R)*-[^11^C]PK11195 images at the level of the knees **(A-C)** with corresponding TAC of arthritic and contralateral knees **(D-F)**. Results are presented as mean ± SD of seven, five and five arthritic rats for [^11^C]DPA-713, [^18^F]DPA-714 and *(R)*-[^11^C]PK11195, respectively. R = right arthritic knee. PET, positron emission tomography; SD, standard deviation; TAC, time-activity curve.

All three TSPO tracers clearly accumulated in arthritic knees (Figure 3A-C). Mean absolute uptake (in SUV) of [^11^C]DPA-713 and [^18^F]DPA-714 in arthritic knees was markedly higher than in contralateral knees (2.37 ± 0.28 versus 1.26 ± 0.16, *P* = 0.02; 1.74 ± 0.46 versus 1.08 ± 0.19, *P* = 0.06, respectively) (Figure 3B,C). For comparison, uptake values (in SUV) of *(R)*-[^11^C]PK11195 in arthritic and contralateral knees were 2.32 ± 0.31 and 1.39 ± 0.17, respectively (Figure 3A). Knee TAC of arthritic and contralateral knees showed that increased [^11^C]DPA-713, [^18^F]DPA-714 and *(R)*-[^11^C]PK11195 uptake in the arthritic knee was evident almost immediately after intravenous injection of the tracer and persisted for at least one hour (Figure 3D-F). This resulted in stable arthritic-to-contralateral knee ratios over time. Increased *(R)*-[^11^C]PK11195, [^11^C]DPA-713 and [^18^F]DPA-714 uptake was further noticed in bone (marrow), heart, lung, spleen, kidney and intestine (see Additional file 2: Figure S2) [24].

### *Ex vivo* tissue distribution and blocking studies

**Figure 4:**
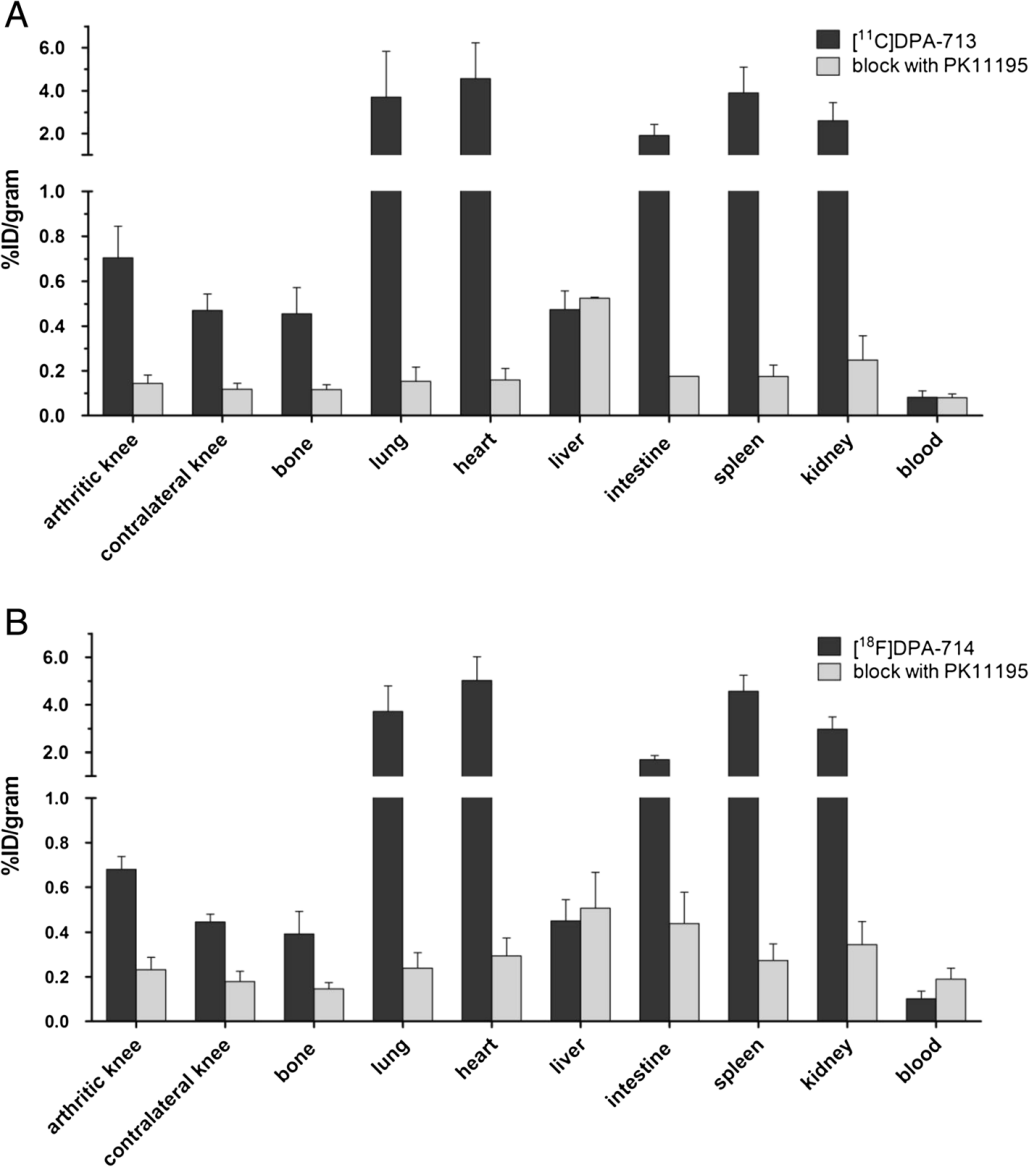
**Tissue distribution of [**^**11**^**C]DPA-713 and [**^**18**^**F]DPA-714 with and without blocking of TSPO binding with unlabelled PK11195.** Tissue distribution of **(A)** [^11^C]DPA-713 and **(B)** [^18^F]DPA-714 in arthritic rats with (grey bars) and without (black bars) blocking of TSPO binding with 5 mg/kg unlabelled PK11195. Results are presented as mean ± SD of seven arthritic rats for [^11^C]DPA-713, six arthritic rats for [^18^F]DPA-714 and two arthritic rats per tracer for blocking experiments. SD, standard deviation; TSPO, translocator protein.

[^11^C]DPA-713 and [^18^F]DPA-714 uptake (expressed as mean %ID/g) in arthritic knees was significantly higher than in contralateral knees (0.70 ± 0.14 versus 0.47 ± 0.07, *P* = 0.02; 0.68 ± 0.06 versus 0.45 ± 0.03, *P* = 0.03, respectively) (Figure 4) and normal knees (0.52 ± 0.05, *P* = 0.05; 0.47 ± 0.03, *P* = 0.004, respectively). Absolute joint uptake in arthritic and contralateral knees of both DPA tracers was comparable with that of *(R)*-[^11^C]PK11195 in arthritic (0.78 ± 0.05) and contralateral (0.57 ± 0.05) joints. Consistently, mean arthritic-to-contralateral knee uptake ratios of [^11^C]DPA-713 (1.49 ± 0.08) and [^18^F]DPA-714 (1.40 ± 0.30) were similar to that of *(R)*-[^11^C]PK11195 (1.40 ± 0.10).

**Figure 5:**
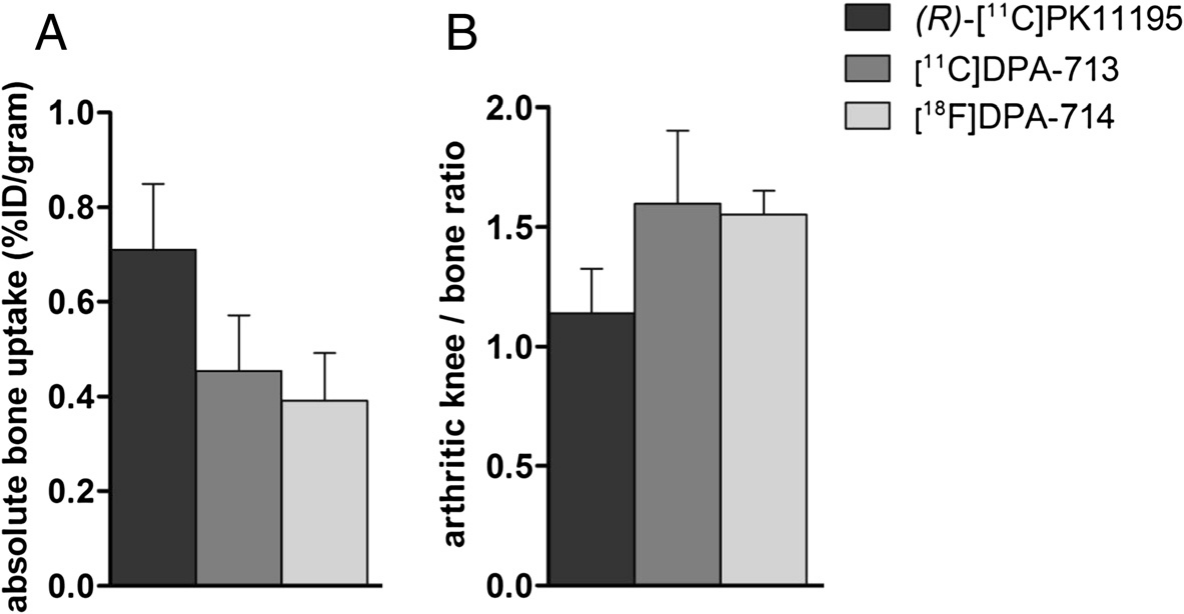
***(R)*-[**^**11**^**C]PK11195, [**^**11**^**C]DPA-713 and [**^**18**^**F]DPA-714 bone uptake.** Absolute uptake **(A)** and arthritic knee-to-bone ratios **(B)** of [^11^C]DPA-713 (dark grey bars) and [^18^F]DPA-714 (light grey bars) compared with that of *(R)*-[^11^C]PK11195 (black bars) obtained from *ex vivo* tissue distribution studies. Results are presented as mean ± SD of five arthritic rats for *(R)*-[^11^C]PK11195, seven arthritic rats for [^11^C]DPA-713 and six arthritic rats for [^18^F]DPA-714. SD, standard deviation.

In previous studies in RA patients, background uptake of *(R)*-[^11^C]PK11195 in peri-articular bone/bone marrow was shown, which potentially hampered the evaluation of subtle joint uptake [3]. Therefore, we examined bone uptake of [^11^C]DPA-713 and [^18^F]DPA-714 in the present study and calculated arthritic knee-to-bone ratios. Significantly higher uptake in bone was found for *(R)*-[^11^C]PK11195 than for [^11^C]DPA-713 and [^18^F]DPA-714 (Figure 5A). Consequently, arthritic knee-to-bone ratios of [^11^C]DPA-713 and [^18^F]DPA-714 were significantly higher than that of *(R)*-[^11^C]PK11195 (Figure 5B). Confirming the PET results, tissue distribution showed accumulation of all TSPO tracers in heart, lung, spleen, kidney and intestine (Figure 4).

Blocking of TSPO binding by pre-administration of unlabelled PK11195 largely abolished uptake of [^11^C]DPA-713 and [^18^F]DPA-714 in these tissues as shown in PET and by tissue distribution studies (see Additional file 2: Figure S2 and Figure 4, respectively). With respect to arthritic knees, mean [^11^C]DPA-713 and [^18^F]DPA-714 uptake (in %ID/gram) in the arthritic knee was five-fold (0.14 ± 0.04) and three-fold (0.23 ± 0.06) decreased, respectively, after blocking (Figure 4).

## Discussion

In this study, the potential of the two high affinity TSPO PET tracers [^11^C]DPA-713 and [^18^F]DPA-714 for imaging activated macrophages in arthritis was evaluated using an mBSA-induced rat model of RA.

*In vitro* binding competition studies confirmed high (relative) binding of both [^11^C]DPA-713 and [^18^F]DPA-714 to TSPO. Furthermore, both DPA tracers showed high uptake in arthritic knee joints of rats as shown by both *in vivo* PET scans and *ex vivo* tissue distribution studies. Uptake in arthritic joints was, at least for a large part, specific, as it was reduced significantly following pre-treatment with an excess of PK11195. Finally, compared with *(R)*-[^11^C]PK11195, bone/bone marrow uptake of both DPA tracers was significantly lower, providing enhanced contrast.

Previous studies have shown higher binding affinities of DPA-713 and DPA-714 to TSPO than PK11195 using rat kidney membrane binding assays [13]. In the present study, TSPO binding of DPA-713 and DPA-714 was compared with that of PK11195 using competition binding studies with [^3^H]DPA-713 in viable human monocytic/macrophage THP-1 cells and CD14^+^ monocytes as a model for *in vivo* macrophages. These studies confirmed increased binding affinities of the DPA tracers compared with PK11195.

PET studies with [^11^C]DPA-713 and [^18^F]DPA-714 in rat models of neuroinflammation have shown reduced nonspecific binding in comparison with *(R)*-[^11^C]PK11195, which would facilitate better detection of mild inflammation [25,26]. For imaging of RA, uptake of the tracer in the inflamed synovium in relation to uptake in surrounding bone and bone marrow is the most important parameter, as the latter may mask subtle uptake in the synovium and can prohibit its precise quantification. In fact, Kam *et al*. [27] showed TSPO expression in rodent bone tissue. In addition, dosimetry studies of *(R)*-[^11^C]PK11195 and [^11^C]DPA-713 showed uptake in human red marrow [28,29]. Therefore, prevention of uptake of TSPO tracers in peri-articular bone of joints affected by RA does not seem to be feasible. Nevertheless, in the present study, both [^11^C]DPA-713 and [^18^F]DPA-714 showed excellent uptake in mBSA-induced arthritis in rats and exhibited markedly lower uptake in peri-articular bone/bone marrow than *(R)*-[^11^C]PK11195. This offers new opportunities for detection of more subtle (sub)clinical arthritis, which is relevant for early diagnosis and therapy monitoring of RA. Future studies should demonstrate whether this holds true for imaging of arthritis in RA patients.

The mBSA-induced rat arthritis model is a widely used model of RA because of its resemblance to human RA pathology at the joint level [17,19,20]. Immunohistochemical studies demonstrated the presence of abundant numbers of TSPO-positive macrophages and neutrophils in the arthritic knee joint. This indicated that TSPO radioligands do not solely target macrophages, but rather the mixed cellular influx in the arthritic knee, reminiscent of human RA.

Unfortunately, the small size of the (arthritic) rat knee joint and small volume of (inflamed) synovium hampers exact determination of tracer uptake in synovial tissue. For this reason, analysis of tracer uptake in PET scans was performed by manually drawing ROIs on top of the whole knee region, because a ROI covering only synovial tissue was not feasible due to insufficient spatial resolution. For determination of *ex vivo* tissue distribution, radioactivity present in the knee joint as a whole, including not only synovium but also peri-articular tissues, was measured. Consequently, both absolute tracer uptake in synovium and arthritic knee-to-bone ratios may have been underestimated.

Finally, for future clinical [^11^C]DPA-713 and [^18^F]DPA-714 studies in RA patients, screening for *TSPO* genetic polymorphism will be important. As previously has been shown in human brain studies, genetic polymorphism in the *TSPO* gene causes differences in binding affinity of all TSPO ligands that are in current clinical use, except *(R)*-[^11^C]PK11195 [30,31]. Therefore, knowledge of patient binding status will be essential for correct quantification of TSPO expression in arthritic joints with PET.

## Conclusions

The two new generation TSPO ligands DPA-713 and DPA-714 showed significantly higher relative binding to TSPO compared to the established TSPO ligand PK11195 in *in vitro* studies. *In vivo*, [^11^C]DPA-713 and [^18^F]DPA-714 proved to be promising radioligands for targeting arthritis in an mBSA-induced arthritis model in rats. While absolute uptake of both tracers in inflamed joints was comparable to that of *(R)*-[^11^C]PK11195, background uptake in bone tissue was significantly lower, potentially providing improved target-to-background contrast on PET images.

## Abbreviations

ID: injected dose; mBSA: methylated bovine serum albumin; PBS: phosphate-buffered saline; PET: positron emission tomography; RA: rheumatoid arthritis; ROI: region of interest; SD: standard deviation; SEM: standard error of the mean; SUV: standardized uptake value; TAC: time-activity curve; TSPO: translocator protein.

## Competing interests

The authors declare that they have no competing interests.

## Authors’ contributions

YG and KW made substantial contributions to acquisition, analysis and interpretation of the data and drafted the manuscript. CM, GJ, AL and CL participated in the design of the study, made substantial contributions to interpretation of the data and critically revised the manuscript. AW coordinated the design and manufacturing of the PET tracers, helped with drafting of the manuscript and made substantial contributions to revision of the manuscript. MH helped with drafting of the manuscript and made substantial contributions to the data analysis of the PET scans and revision of the manuscript. MK made substantial contributions to design of the study and revision of the manuscript. All authors read and approved the final manuscript.

## Supplementary Material

Additional file 1 **Synthesis of [**^11^C]DPA-713, [^18^F]DPA-714 and [^3^H]DPA-713. Detailed synthesis protocols of [^11^C]DPA-713, [^18^F]DPA-714 and [^3^H]DPA-713 including reaction schemes. Click here for file

Additional file 2: Figure S2 [^11^C]DPA-713 and [^18^F]DPA-714 PET images with and without blocking of TSPO binding with unlabelled PK11195. Representative [^11^C]DPA-713 (A) and [^18^F]DPA-714 (B) PET images of arthritic rats without (left) and with (right) blocking of TSPO binding with PK11195. SUV = standardized uptake value. Click here for file
