# Supplementary material for: Promising potential of new generation translocator protein tracers providing enhanced contrast of arthritis imaging by positron emission tomography in a rat model of arthritis
Source: Arthritis Res Ther. 2014 Mar 14;16(2):R70. doi: 10.1186/ar4509 (PMC4060541; doi:10.1186/ar4509)
Supplement: Additional file 1 — Synthesis of [11C]DPA-713, [18F]DPA-714 and [3H]DPA-713. Detailed synthesis protocols of [11C]DPA-713, [18F]DPA-714 and [3H]DPA-713 including reaction schemes. [file ar4509-S1.pdf]

Additional file 1. Synthesis of [^{18}F]DPA-714, [^{11}C]DPA-713 and [^{3}H]DPA-713

Synthesis of [^{18}F]DPA-714

The radionuclide, fluor-18, was produced by the (p,n) nuclear reaction onto oxygen-18 enriched (>95%) water in a niobium target holder with 18 MeV protons generated by a Cyclone 18/9 (IBA, Louvain-la-Neuve, Belgium). After irradiation, [^{18}F]fluoride was trapped on a Machery Nagel PS-HCO_3 anion exchange column (ABX, Radeberg, Germany). It was eluted from the anion exchange column into a screw cap reaction vessel with 1 mL of acetonitrile/water (9/1, v/v) containing 13 mg (34.5 µmol) of Kryptofix 2.2.2 (Merck Millipore, Billerica, USA) and 2 mg (14.5 µmol) of potassium carbonate (Aldrich, Zwijndrecht, The Netherlands). The solution was evaporated to dryness under a helium flow (50 mL/min) and reduced pressure (10-15 hPa) at 90 °C. Acetonitrile (0.5 mL, < 10 ppm water, Merck Millipore, Billerica, USA) was added and evaporated again to remove any residual water. Next the precursor, toluene-4-sulfonic acid 2-[4-(3-diethylcarbamoylmethyl-5,7-dimethyl-pyrazolo-[1,5-a]pyrimidin-2-yl)-phenoxy]-ethyl ester (**1**, 4 mg, synthesized at University of Sydney, [1]), dissolved in 500 µL of acetonitrile was added to the reaction vessel (Scheme 1). The mixture was heated for 10 minutes at 105 °C and subsequently diluted with 3.0 mL of water. The total solution was subjected to HPLC purification with a XTerra RP18 5 µm, 250x10 mm and 0.1M ammonium acetate/acetonitrile 65/35 as eluent at a flow rate of 4 mL/min. The product eluted at around 21 minutes and was collected in 40 mL of water. The total solution was passed over a Sep-Pak tC18 (Waters, Etten-Leur, The Netherlands) and subsequently the Sep-Pak was washed with 20 mL of sterile water for injection (B. Braun, Sempach, Switzerland). The product was collected by elution of the Sep-

Pak with 1.0 mL of sterile ethanol (96%, pharmacy VUmc, Amsterdam, The Netherlands) and diluted with sterile 0.9% sodium chloride solution (B. Braun, Sempach, Switzerland). The final solution was passed over a sterile Millex GV 0.22 µm filter (Merck Millipore, Billerica, USA). Radiochemical purity was assessed with HPLC on a Chromolith performance RP18e 4,6*100mm and water/acetonitrile 70/30 as eluent at a flow rate of 4.0 mL/min and was >95%. Specific activity was > 75 GBq/µmole and no UV impurities were observed on HPLC. The decay corrected radiochemical yield was 19-43% calculated from [^{18}F]fluoride.

Scheme 1

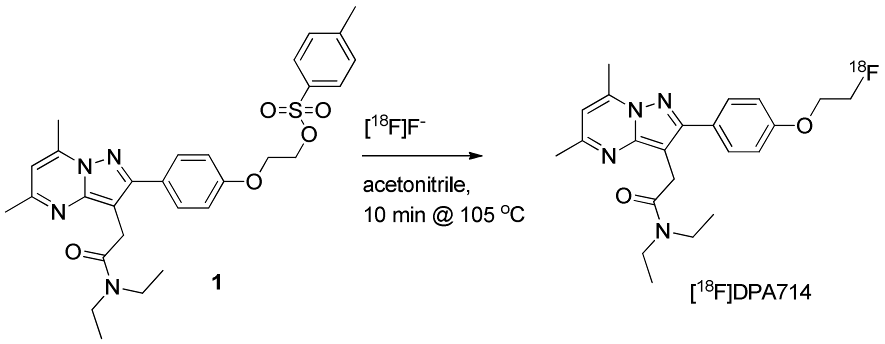

Synthesis of [^{11}C]DPA-713

The radionuclide, carbon-11, was produced by the (p,□) nuclear reaction onto natural nitrogen gas (5.0) which contained 0.5% oxygen (5.0) aluminium target holder with 15 MeV protons generated by a Cyclone 18/9 (IBA, Louvain-la-Neuve, Belgium). After irradiation, carbon-11 was isolated as [^{11}C]CO_2 and trapped in a stainless steel coil dispersed in liquid nitrogen. Subsequently the [^{11}C]CO_2 was transferred to the hotcel with a 10 mL/min He gas flow where it was diverted into a reaction vessel containing 100 µL of a 0.1 M solution of $LiAlH_4$ in THF (ABX, Radeberg, Germany). After all [^{11}C]CO_2 was collected in the reaction vessel, the THF was evaporated by rising the temperature to 130 °C while blowing 20 mL/min with helium. When the temperature of 130 °C was reached, all THF was evaporated and 200 µL of HI (57%, Merck Millipore, Billerica, USA) was added to yield [^{11}C]methyl iodide which was distilled from the reaction mixture by a 20 mL/min helium flow into a second reaction vessel containing the precursor (**2**, synthesized at University of Sydney, [2]) dissolved in 250 µL of DMF (Aldrich, Zwijndrecht, The Netherlands) and 4 µL of a 3 M solution of NaOH in water as a base (Scheme 2). After heating the mixture for 5 minutes at 80 °C the mixture was cooled to 25 °C and injected onto a semi-preparative HPLC system (column: reprosphere C18-DE 5µ 50x8 mm with 60/40 ACN/water + 0.1% diisopropylethyl amine at 3 ml/min for purification. The product, [^{11}C]DPA713 eluted at 8 minutes and was separately collected into a 50 mL of water. This solution was passed over Sep-Pak tC18 (Waters, Etten-Leur, The Netherlands) and the Sep-Pak was subsequently washed with 20 mL of sterile water (B. Braun, Sempach, Switzerland). The product was eluted with 1.0 mL of sterile ethanol (96%, VUmc pharmacy, Amsterdam, The Netherlands) and dilutes with a sterile 7.01 mM solution of NaH_2PO_4 in saline (VUmc pharmacy, Amsterdam, The Netherlands) and filtered over a sterile Millex GV 0.22 µm filter

(Merck Millipore, Billerica, USA). The radiochemical purity of [^{11}C]DPA713 was assessed by HPLC (column XTerraC-18 MS 150x3.6 mm 5 µm, eluent: NH_4HPO_4 (pH 2.5) / Acetonitrile 50/50 as eluent at 1 mL/min) and was shown to be >98% radiochemically pure, no UV impurities were observed on HPLC. The specific activity of [^{11}C]DPA713 was > 40 GBq/µmole and the decay corrected radiochemical yield calculated from [^{11}C]CO_2 was 23-28%

Scheme 2:

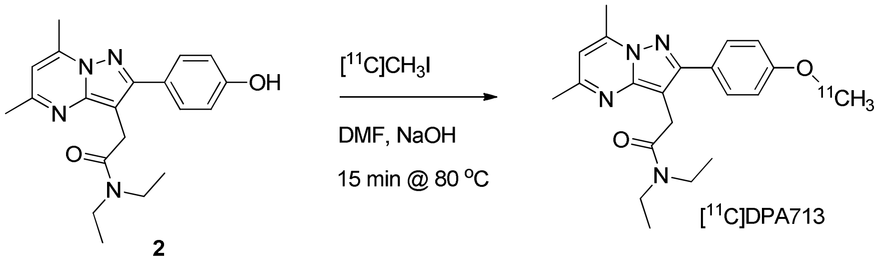

Synthesis of [^{3}H]DPA-713

The tritium labelled analog of DPA713 was synthesized from **2** according to the same procedure as for [^{11}C]DPA714, but with [^{3}H]methyl nosylate (solution in hexanes, Perkin Elmer, Waltham, USA) instead of [^{11}C]CH_3I (Scheme 3). The methylation reaction was elongated to 15 minutes and the product was isolated in 1.5 mL of 96% of ethanol from the Sep-Pak and stored at -20 °C. Radiochemical purity was 99.8%, no UV impurities were observed and specific activity was 2.37 MBq/nmole, radiochemical yield 38%.

Scheme 3.

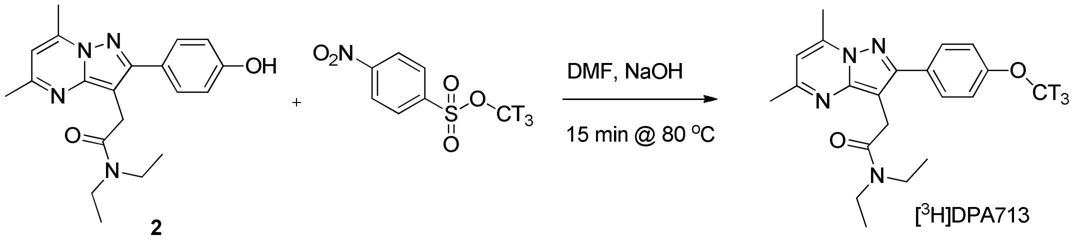

References

1. James ML, Fulton RR, Vercoullie J, Henderson DJ, Garreau L, Chalon S, Dolle F, Costa B, Guilloteau D, Kassiou M: **DPA-714, a new translocator protein-specific ligand: synthesis, radiofluorination, and pharmacologic characterization.** *J Nucl Med* 2008, **49:**814-822.

2. James ML, Fulton RR, Henderson DJ, Eberl S, Meikle SR, Thomson S, Allan RD, Dolle F, Fulham MJ, Kassiou M: **Synthesis and in vivo evaluation of a novel peripheral benzodiazepine receptor PET radioligand.** *Bioorg Med Chem* 2005, **13:**6188-6194.
