# Supplementary figures and images for: Promising potential of new generation translocator protein tracers providing enhanced contrast of arthritis imaging by positron emission tomography in a rat model of arthritis
Source: Arthritis Res Ther. 2014 Mar 14;16(2):R70. doi: 10.1186/ar4509 (PMC4060541; doi:10.1186/ar4509)

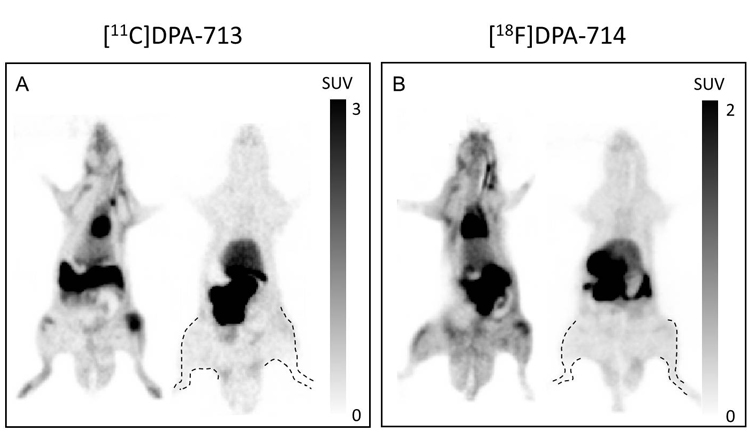

Supplement: Additional file 2: Figure S2 — [11C]DPA-713 and [18F]DPA-714 PET images with and without blocking of TSPO binding with unlabelled PK11195. Representative [11C]DPA-713 (A) and [18F]DPA-714 (B) PET images of arthritic rats without (left) and with (right) blocking of TSPO binding with PK11195. SUV = standardized uptake value. [file ar4509-S2.jpeg]
